# A long way to go – Estimates of combined water, sanitation and hygiene coverage for 25 sub-Saharan African countries

**DOI:** 10.1371/journal.pone.0171783

**Published:** 2017-02-09

**Authors:** Rachel Roche, Robert Bain, Oliver Cumming

**Affiliations:** 1 London School of Hygiene and Tropical Medicine, Department of Disease Control, Faculty of Infectious and Tropical Diseases, London, United Kingdom; 2 Division of Data, Research and Policy, UNICEF, New York, New York, United States of America; Leibniz Institute for Prevention Research and Epidemiology BIPS, GERMANY

## Abstract

**Background:**

Water, sanitation and hygiene (WASH) are essential for a healthy and dignified life. International targets to reduce inadequate WASH coverage were set under the Millennium Development Goals (MDGs, 1990–2015) and now the Sustainable Development Goals (SDGs, 2016–2030). The MDGs called for halving the proportion of the population without access to adequate water and sanitation, whereas the SDGs call for universal access, require the progressive reduction of inequalities, and include hygiene in addition to water and sanitation. Estimating access to complete WASH coverage provides a baseline for monitoring during the SDG period. Sub-Saharan Africa (SSA) has among the lowest rates of WASH coverage globally.

**Methods:**

The most recent available Demographic Household Survey (DHS) or Multiple Indicator Cluster Survey (MICS) data for 25 countries in SSA were analysed to estimate national and regional coverage for combined water and sanitation (a combined MDG indicator for ‘improved’ access) and combined water with collection time within 30 minutes plus sanitation and hygiene (a combined SDG indicator for ‘basic’ access). Coverage rates were estimated separately for urban and rural populations and for wealth quintiles. Frequency ratios and percentage point differences for urban and rural coverage were calculated to give both relative and absolute measures of urban-rural inequality. Wealth inequalities were assessed by visual examination of coverage across wealth quintiles in urban and rural populations and by calculating concentration indices as standard measures of relative wealth related inequality that give an indication of how unevenly a health indicator is distributed across the wealth distribution.

**Results:**

Combined MDG coverage in SSA was 20%, and combined basic SDG coverage was 4%; an estimated 921 million people lacked basic SDG coverage. Relative measures of inequality were higher for combined basic SDG coverage than combined MDG coverage, but absolute inequality was lower. Rural combined basic SDG coverage was close to zero in many countries.

**Conclusions:**

Our estimates help to quantify the scale of progress required to achieve universal WASH access in low-income countries, as envisaged under the water and sanitation SDG. Monitoring and reporting changes in the proportion of the national population with access to water, sanitation *and* hygiene may be useful in focusing WASH policy and investments towards the areas of greatest need.

## Introduction

In 2015 the Millennium Development Goal (MDG) period (1990–2015) came to an end. The MDG target for water and sanitation (7c), ‘to halve the proportion of people without sustainable access to safe drinking-water and basic sanitation’ was declared to have been met in 2010 for drinking water, while the target for sanitation was missed [1]. The new Sustainable Development Goals (SDGs) were endorsed by the UN General Assembly in September 2015, setting ambitious new goals and targets for 2030. SDG goal 6, to ‘ensure availability and sustainable management of water and sanitation for all’ reflects substantially increased ambition for improving access to the unserved as it is now a goal of universal access, requiring the progressive reduction of inequalities and including hygiene in addition to water and sanitation [2]. The inclusion of such targets reflects a recognition of central importance of basic water, sanitation and hygiene (WASH) for a healthy and dignified life, and the ratification of the human right to drinking water and sanitation in 2010 [3].

The focus on progressive realisation brings a greater need for disaggregated monitoring that identifies inequities in access in order to support targeting interventions towards underserved populations. Ongoing inequalities in access to both water and sanitation by setting (rural and urban) and by wealth quintiles were identified during progress monitoring for the MDGs [1]. Understanding patterns of inequalities in WASH access and how these are affected by the different criteria used to define access for the SDGs is important in order to provide a meaningful baseline against which progress during the SDG period can be assessed.

Global progress monitoring towards the MDGs was undertaken through the Joint Monitoring Programme (JMP) of the World Health Organisation (WHO) and United Nations Children’s Fund (UNICEF). The JMP used nationally representative household survey and census data including Demographic and Health Surveys (DHS) and Multiple Indicator Cluster Surveys (MICS) to monitor progress. Although target 7c included both water and sanitation, the JMP reported on use of improved water and improved sanitation separately, and target achievement at the end of the MDGs was based on a separate assessment for water and sanitation [1]. The JMP reported a combined estimate in 2012 based on analysis of data from 59 developing countries [4] and found that 50% of the population had both improved water and sanitation, compared with 75% that had improved water and 59% that had improved sanitation. Comparable data on hygiene have been collected since 2009, when a standardised handwashing module was created and added to MICS and DHS surveys [1, 5, 6]. Prior to this, handwashing data were collected in a variety of ways, such that data was not comparable between countries [7]. In later years of MDG monitoring the binary classification of facilities as ‘improved’ or ‘unimproved’ was expanded and a ‘service ladder’ approach was developed, in which piped water on premises and improved, unshared sanitation are the highest service levels, and surface water and open defecation the lowest. The new SDG indicators perpetuate separate monitoring of water and sanitation. Hygiene was not included in the MDG targets, but is included in the SDGs within the sanitation target 6.2: ‘By 2030, achieve access to adequate and equitable sanitation and hygiene for all and end open defecation, paying special attention to the needs of women and girls and those in vulnerable situations’ [2]. The JMP proposes to build upon the service ladder approach for SDG monitoring, with a disaggregation of ‘improved’ services into ‘basic’ and ‘safely managed’ water and sanitation, with safely managed services requiring the safe management of excreta and monitoring of water quality, accessibility and availability [8]. Monitoring this higher service level will require additional information sources in some countries and is proposed as a short to medium-term goal. For SDG monitoring ‘basic’ water also requires that the time taken for collection should not be more than 30 minutes. The SDGs maintain separate targets for access to safe and affordable drinking water (target 6.1) and to adequate and equitable sanitation and hygiene (target 6.2) and do not report the proportion of households that have all three.

The consequences of poor access to WASH remain profound: inadequate WASH remains the most significant contributor to the global burden of diarrhoeal disease [9], with an estimated 842,000 diarrhoea deaths attributable to this cluster of risk factors in 2012 [10, 11]. The impact of diarrhoea is most acute in children under five and it remains a leading cause of child mortality worldwide [12], and may be an important determinant of childhood undernutrition [13, 14]. Inadequate WASH is also associated with substantially increased maternal mortality [15] as well as the transmission of a range of neglected tropical diseases [16–19] and respiratory infections [20, 21]. Inadequate access to WASH also has significant economic, environmental and social impacts. Travel and waiting time has an opportunity cost that impacts on GDP as time is not able to be spent on other activities that could contribute to economic growth [22]. Improved WASH has a role in reducing gender inequality and promoting women’s economic empowerment [23]; the burden of water collection falls predominantly on women [24], and women are more vulnerable to attacks and sexual assault when using public sanitation facilities. Improved sanitation facilities in schools can improve attendance and this too may be more important for girls due to the importance of menstrual hygiene management [25].

Sub-Saharan Africa (SSA) has among the lowest levels of access to both drinking water and sanitation globally. SSA failed to meet the MDG target for drinking water, with 32% of the population estimated not to have access to an improved water source at the end of the MDG period [1], and an estimated 102 million people still using surface water. SSA also has the lowest regional rate of coverage of improved sanitation, with an estimated 695 million people still using unimproved facilities [1]. Whilst estimating hygiene practice is challenging, the prevalence of handwashing with soap at critical times (after defecation and before eating) for SSA has been estimated to be just 14% [26]. Beyond these regional figures, large ongoing disparities in WASH access are known to exist between urban and rural populations and between the rich and poor within countries [1].

Faecal-oral transmission of diarrhoea occurs by multiple inter-linked pathways as depicted by Wagner and Lanoix’s [27] F-Diagram, with five transmission routes for faecal-oral diseases: fluids (or water); fields (or soil); flies; fingers; and food. From this framework it follows that combined WASH interventions that interrupt multiple pathways of transmission are likely to be more effective than interventions that address fewer transmission pathways [28]. However, several reviews in the 2000s on the evidence for the effectiveness of WASH interventions in reducing diarrhoea transmission did not find any evidence of an additional effect from combined interventions [29–31]. A subsequent systematic review in 2010 [32] highlighted the poor quality and poor description of many of the available studies; in particular all studies previously categorised as ‘sanitation’ interventions in fact included an intervention to improve water supply and/or hygiene. One more recent analysis though has included an additional effect of combined interventions, estimating a risk ratio of 0.88 (CI 0.77, 1.01) for the additional effect of combining a water intervention with either hygiene education and/or improved sanitation [10]. The effect of interventions to promote handwashing with soap on reducing diarrhoea have consistently been found to be strong [20, 26, 32–34], although in one review that applied a standard adjustment for non-blinding the effect of handwashing became non-significant, but the point estimate was substantial (RR 0.77, 95% CI 0.32–1.86) [26]. The most recent systematic review found a rate ratio for diarrhoea episodes of 0.72 (95% CI 0.62–0.83) for community interventions in developing countries, with a larger effect size in trials where soap was provided (rate ratio 0.66, 95% CI 0.56–0.78) [34].

Although the epidemiological evidence does not provide a clear picture of the additional impact of combined water, sanitation and hygiene interventions, it is clear that the optimum scenario is for all three to be in place and that hygiene in particular is a valuable, yet overlooked, dimension of WASH access.

The aims of this study are to estimate the prevalence of ‘combined MDG coverage’ (water and sanitation) and ‘combined basic SDG coverage’ (water with collection time under 30 minutes plus sanitation and hygiene) in SSA, and to investigate how these vary by urban/rural location and socio-economic status. Using the latest available DHS or MICS survey data, our objectives were to: (i) estimate the proportion of the population with combined MDG coverage and combined basic SDG coverage for 25 individual SSA countries and for the SSA region; (ii) estimate the proportion of these populations with combined MDG and combined basic SDG coverage by urban/rural location and wealth quintile.

## Methods

### 2.1 Data sources

This study uses nationally representative household survey data that was used by the JMP to monitor global progress towards the MDGs and currently used to monitor progress towards the SDGs. We used national datasets for SSA countries drawn from two survey programmes: DHS and MICS. These data are publicly available and were accessed via their respective websites: www.dhsprogram.com and http://mics.unicef.org. Countries included in the analysis met two criteria: (i) part of the SSA region, as defined by the UN MDG regional groupings [35]; (ii) a DHS or MICS dataset was available which included standardised questions on handwashing (details below). Datasets were available for a total of 25 countries from the SSA region. All datasets were from years 2010–2014. Where multiple datasets were available for the same country, the most recent survey was used.

### 2.2 Definitions of access

#### 2.2.1 Definitions of improved and unimproved water and sanitation facilities

Definitions of ‘improved’ water and sanitation were used as defined by the JMP for the MDG targets, with certain adjustments made (Table 1).

**Table 1:** Definitions of improved and unimproved facilities. All criteria for multiple questions or observations must be met in order for facilities to be defined as improved [39]

MDG Criteria		
1. Water		
Question	Improved	Unimproved
What is the main source of drinking water for members of your household?	• Piped into dwelling • Piped to yard/plot • Public tap/standpipe • Tube well/borehole • Protected dug well • Protected spring • Rainwater • Piped to neighbour • Bottled water (if improved water used for other purposes cooking / handwashing)	• Unprotected well • Unprotected spring • Tanker truck • Cart with small tank • Surface water (river / dam / lake / pond / stream / canal / irrigation channel) • Bottled water (if improved water not used for other purposes cooking / handwashing) • Sachet water • Other
What is the main source of water your household uses for other purposes such as cooking and cleaning?(Only relevant if bottled water is the main source of drinking water)	• Piped into dwelling • Piped into yard/plot • Public tap/standpipe • Tube well/borehole • Protected well • Protected spring • Rainwater	• Unprotected well • Unprotected spring • Surface water • Tanker truck • Cart with small tank • Other
2. Sanitation		
Questions	Improved	Unimproved
What kind of toilet facility do members of your household usually use?	• Flush or pour flush toilet flush to piped sewer system • Flush to septic tank • Flush to pit latrine • Ventilated improved pit latrine • Pit latrine with slab • Composting toilet • Flush, don’t know where	• Flush to somewhere else • Pit latrine without slab / open pit • Bucket toilet • Hanging toilet / hanging latrine • No facility / bush / field • Mobile toilet • Other
Do you share this facility with others who are not members of your household?	• Facility not shared with other households	• Facility shared with other households
3. Combined MDG = (1) + (2)		
SDG Criteria		
4. Water		
Question	Improved	Unimproved
How long does it take you to go, collect water and come back?	• On premises or 0–30 minutes	• >30 minutes
5. Handwashing		
Observations	Improved	Unimproved
Please show me where members of your household most often wash their hands.	• Handwashing facility observed	• Handwashing facility not observed (not in dwelling / yard / plot, no permission to see or other reason)
Observation only: observe presence of water at the place for hand washing.	• Water is available	• Water is not available
Observation only: observe presence of soap, detergent, or other cleansing agent at the specific place for hand washing	• Soap or detergent (bar, liquid, powder, paste) is present	• No soap or detergent
6. Combined basic SDG = (3) + (4) + (5)		

For drinking water, the JMP definition states that bottled water is only considered improved if an improved source is also used for other purposes such as cooking and handwashing; however, in practice information on the secondary source of water was only available in 7 of the 25 datasets. Where this data was not available we classified bottled water as an improved source, as this was how it had been categorised in the JMP estimates for these countries [36], although in practice this accounted for only 1% of respondents.

For sanitation, in the JMP country files [37] the sanitation category ‘flush, don’t know where’ was classified as improved in all country datasets where it was present, so this was categorised as improved in order to more closely follow the JMP estimates. In addition the JMP method estimates the proportion of shared sanitation facilities from an average of available ratios of shared facilities from household surveys and censuses and subtracts this from the total to give an estimate of unshared facilities. However as information on whether the sanitation facility was shared was available within datasets used for this analysis, this was used to directly class shared facilities as unimproved. Lastly, where there were missing values for variables used in the analysis these were dropped. This accounted for between 0 and 2% of observations in individual datasets, or 0.5% of observations overall.

Where countries had additional or different categories of water sources or sanitation facilities to those in the JMP definition, the country’s published survey reports were used to check definitions and these were cross-checked against the proportions reported in the JMP country files. Details of the classifications of additional facility classes that did not clearly fit into the JMP definitions are given in S1 Table.

#### 2.2.2 Definitions of combined MDG and combined basic SDG access

‘Combined MDG’ access was defined for the analysis as having both improved water and improved sanitation under the above definitions.

The SDGs have more ambitious definitions of improved access than the MDGs, and propose a disaggregation of improved’ services into ‘basic’ and ‘safely managed’ water and sanitation, with safely managed services requiring the safe management of excreta and monitoring of water quality, accessibility and availability [8]. Here we consider ‘basic’ SDG coverage as there is as yet insufficient data to assess coverage of safely managed services. The criteria for basic SDG coverage are more stringent than the MDGs in two ways:

1. The SDG target for water specifies that the time it takes for a round trip to collect water must be less than 30 minutes.
2. The SDG target for sanitation specifies that this must also include a handwashing facility, as defined in indicator 6.2.1: ‘percentage of population using safely managed services, including a hand-washing facility with soap and water’ [38].

‘Combined SDG’ access was therefore defined for the analysis as having both improved water with a collection time of under 30 minutes, plus sanitation and a handwashing facility with soap.

### 2.3 Data analysis

All statistical analyses were conducted using Stata 14 [40]. The *svyset* command was used to account for the complex survey sampling methods used in the original surveys (clustering, stratification and sample weights). As survey data on water, sanitation and hygiene is collected at a household level, the household response was allocated to the number of *de jure* household members to estimate for population coverage.

#### 2.3.1 National and regional estimates

Estimates for each country and their 95% confidence intervals (CI) were first made separately for urban and rural populations as per published JMP methods [41]. Separate estimates were made for the proportion of the population having improved water, improved sanitation, and improved hygiene. Combined estimates were made for the proportion having both improved water and improved sanitation (combined MDG indicator); combined water, sanitation and hygiene; and combined water, sanitation and hygiene corrected for classifying water as improved only where collection time was under 30 minutes (combined SDG indicator).

As per published JMP methods [41] after the separate urban and rural estimates were made country totals were calculated as a population-weighted average of the urban and rural numbers using the 2015 population estimates of the UN Population Division [42]. Regional estimates were made by summing the urban and rural populations for all countries for which data were available and expressing these as a percentage of the total population of those countries. In the JMP method, if the countries for which data is available contain at least 50% of the regional population this proportion can be applied to the regional population and to corresponding sub-populations [39]. In this case the population of the countries for which data were available was 68% of the population of SSA and therefore this approach was used. National and regional 95% CI were calculated as population-weighted averages of individual countries’ urban and rural 95% CI.

Under the JMP method coverage rates for years in which survey data are not available are estimated using a linear regression of the proportions as a function of time [39]. Regression lines are extrapolated for up to two years before or after the earliest or most recent census or survey year, after which the coverage rate at the end of the two-year extrapolation is reported for up to a further four years. For this analysis coverage rates were not extrapolated to provide an estimate for one year, as handwashing data was available for only one year given its recent introduction to the DHS and MICS. Therefore, the estimates provided in this analysis relate to the broad time period of the survey data that were used (2010–2014) and are lower than the final 2015 JMP estimates for water and sanitation.

For subsequent equality analyses, the estimates for the combined MDG indicator and the combined SDG indicator were used. The datasets for two countries, Central African Republic and Chad, did not have information on collection time for water, therefore for these countries the combined water, sanitation and hygiene estimates were used in subsequent analyses and for the regional estimates for the combined SDG indicator. We conducted a Spearman’s rank test in order to assess how the rankings of countries varied across the two indicators.

#### 2.3.2 Sub-national inequalities

We considered two dimensions of inequality: wealth and rural/urban residence. To assess disparities between urban and rural populations we estimated the proportions with access using national definitions of urban and rural households from each survey. To assess how urban–rural inequality varied across the two definitions we considered two measures of inequality between urban and rural coverage: frequency ratios and percentage point differences. We conducted Pearson correlations of these measures to assess the associations of urban–rural inequality across the two indicators.

To analyse inequalities by wealth, we used the wealth indices within the datasets. DHS and MICS wealth indices are calculated using principal components analysis applied to a range of household characteristics [43]. Water, sanitation and, more recently, handwashing facilities have been included as “assets” in the creation of these standard indices, which brings a risk of confounding. A recent sensitivity analysis, however, suggested that asset-based wealth indices that excluded water and sanitation were highly correlated with those that included water and sanitation, such that standard asset indices were sufficient for assessing national level disparities [44].

To assess how coverage varied by wealth, we first created separate urban and rural wealth quintiles for each country from the wealth index score. We then stratified our urban and rural estimates by the wealth quintiles for each country and examined these visually. Visual examination of the coverage across wealth quintiles allows for a crude description of the pattern of inequalities, which can be defined as either ‘top’, ‘linear’ or ‘bottom’ inequality patterns as first used by Victora et al. [45]. Top inequality describes a situation where coverage of the indicator is predominantly within the richest quintile, whereas a bottom inequality pattern shows that coverage in the poorest quintile lags behind the rest. A linear pattern shows that there is a steady change in coverage across the wealth quintiles.

Next, in order to provide a relative measure of wealth-related equality, we calculated urban and rural concentration indices and their 95% confidence intervals (CI) for the indicators using the World Bank’s DASP toolkit for Stata 14 [46]. Concentration indices are a standard measure of wealth related health inequality [47] and give an indication of how unevenly a health indicator is distributed across the wealth distribution. Concentration indices range from -1 to 1, with a value of zero representing equal coverage across the wealth distribution. Negative values indicate that the indicator is disproportionately concentrated among the poor, while positive values represent that the indicator is disproportionately concentrated among the wealthy. In order to facilitate international comparison the concentration indices were normalised to the overall proportion of the country’s urban or rural population with the indicator [48].

### 2.4 Ethics statement

This is a secondary analysis of previously published and publicly available household survey data and was approved by the London School of Hygiene and Tropical Medicine MSc Research Ethics Committee on 13/07/2015 (Ref 9229).

## Results

We analysed the most recent available DHS or MICS data for 25 countries in SSA to estimate national and regional coverage for complete MDG and basic SDG access. We estimated coverage rates separately for urban and rural populations and for wealth quintiles. We calculated concentration indices for urban and rural coverage of each indicator as a measure of relative inequality.

### 3.1 National and regional coverage for MDG and basic SDG access

Coverage for combined MDG improved access (use of both improved water and sanitation facilities as defined under MDG target 7c) varies significantly across the SSA region from 6.7% in Ethiopia to 47.2% in Rwanda, with a regional average of 19.7% (Table 2). Coverage for combined SDG access (use of improved water (with collection time within 30 minutes), sanitation, and hygiene facilities) is much lower, ranging from 0.8% in Liberia to 22.6% in Namibia and with a regional average of just 4.2% across SSA. If the estimate for combined SDG access did not exclude water sources with a collection time of over 30 minutes the estimate would be very similar, with a range from 0.8% in Liberia to 22.8% in Namibia, and an average of 4.4%. Spearman’s rank correlation for combined MDG and combined SDG coverage of individual countries is 0.65 (p<0.001), indicating that overall there is a strong correlation in ranking between the two indicators. However, there are some countries that have substantial changes in rankings between the two indicators. For example, Rwanda has the highest rate of combined MDG access, and yet has one of the lowest rates of combined SDG access, whereas Chad has the third lowest rate of combined MDG coverage and yet a mid-range rate of combined SDG coverage.

**Table 2:** Proportion of population with access to separate and combined WASH facilities, by country and regional.

Country	Survey year	Survey type	Improved water (% (95% CI))	Improved sanitation (% (95% CI))	Improved hygiene (% (95% CI))	Water and sanitation (combined MDG indicator) (% (95% CI))	Water under 30 mins (SDG basic water indicator) (%, (95% CI))	Sanitation and hygiene (SDG basic sanitation indicator) (%, (95% CI))	Water under 30 minutes, sanitation and hygiene (combined SDG indicator) (% (95% CI))	Ratio MDG/SDG coverage	Percentage point difference (MDG-SDG) (% (95% CI))
Benin	2011–2012	DHS	77.7 (75.2–80.1)	17.0 (15.6–18.4)	9.2 (8.2–9.9)	15.7 (14.3–17.1)	73.2 (70.6–75.6)	4.0 (3.5–4.7)	3.8 (3.3–4.5)	4.1	11.8 (9.8–13.8)
Burkina Faso	2010	DHS	79.2 (76.8–81.4)	21.1 (19.2–23.0)	11.6 (10.0–13.5)	20.0 (18.2–22.0)	69. (66.5–71.3)	5.8 (4.7–7.0)	5.5 (4.5–6.7)	3.6	14.5 (11.5–17.5)
Burundi	2010	DHS	78.2 (74.5–81.2)	35.0 (31.5–38.6)	7.1 (5.4–9.1)	27.5 (24.2–31.1)	57.1 (53.5–60.3)	4.0 (2.7–5.8)	3.4 (2.2–5.1)	8.1	24.2 (19.1–28.9)
Central African Republic	2010	MICS	68.1 (64.5–71.5)	23.8 (21.4–26.3)	16.6 (13.9–19.5)	20.5 (18.3–22.9)	*	6.7 (5.2–8.5)	5.9 (4.5–7.6)*	3.5	14.6 (10.7–18.4)
Chad	2010	MICS	54.2 (49.8–58.4)	12.0 (10.3–13.9)	24.1 (21.7–26.7)	10.3 (8.8–12.0)	*	5.9 (5.0–7.0)	5.4 (4.6–6.5)*	1.9	4.9 (2.4–7.5)
Comoros	2012	DHS	87.8 (83.8–90.8)	30.0 (25.8–34.5)	15.7 (12.4–19.5)	27.8 (23.6–32.4)	81.5 (77.4–85.0)	7.2 (5.3–9.6)	6.3 (4.5–8.7)	4.4	21.5 (14.9–27.9)
Côte d'Ivoire	2011–2012	DHS	81.2 (77.2–84.6)	25.3 (21.5–29.3)	16.1 (13.3–19.4)	24.3 (20.6–28.2)	75.5 (71.4–79.1)	9.4 (7.2–12.1)	9.3 (7.1–12.0)	2.6	15.0 (8.6–21.1)
Democratic Republic of the Congo	2013–2014	DHS	57.2 (52.4–61.9)	21.2 (17.8–25.2)	4.5 (3.7–5.6)	13.8 (11.0–17.1)	43.7 (38.7–49.1)	1.9 (1.4–0.5)	1.6 (1.1–2.4)	8.4	12.1 (8.6–16.0)
Ethiopia	2011	DHS	53.4 (49.0–57.6)	9.4 (7.5–11.7)	1.2 (0.6–2.3)	6.7 (5.1–8.7)	36.0 (32.6–39.3)	0.9 (0.4–2.0)	0.9 (0.4–2.0)	7.8	5.9 (3.2–8.4)
Ghana	2011	MICS	64.1 (60.3–67.7)	15.9 (13.7–18.5)	10.2 (8.7–12.0)	8.2 (6.8–9.9)	56.4 (52.9–59.8)	4.6 (3.5–6.0)	2.4 (1.8–3.2)	3.4	5.8 (3.6–8.1)
Guinea	2012	DHS	78.4 (74.2–81.9)	23.6 (20.4–27.0)	7.8 (6.1–9.8)	21.6 (18.6–25.0)	66.2 (61.8–70.3)	4.4 (3.2–6.0)	4.1 (3.0–5.7)	5.3	17.5 (12.9–22.0)
Liberia	2013	DHS	73.0 (65.7–78.5)	16.9 (13.8–20.4)	1.2 (0.7–2.1)	15.6 (12.6–19.1)	64.5 (58.6–70.0)	0.8 (0.5–1.5)	0.8 (0.5–1.5)	19.0	14.7 (11.1–18.7)
Mali	2012–2013	DHS	74.3 (70.6–77.6)	30.2 (26.9–33.6)	13.2 (11.3–15.3)	26.5 (23.5–29.7)	72.1 (68.5–75.4)	7.2 (5.8–8.9)	6.8 (5.5–8.5)	3.9	19.7 (15.0–24.3)
Mauritania	2011	MICS	55.2 (51.3–59.0)	39.7 (36.8–42.6)	34.9 (31.8–38.2)	25.5 (22.8–28.4)	49.8 (45.8–53.9)	17.8 (15.5–20.3)	11.5 (9.7–13.5)	2.2	14.0 (9.3–18.7)
Mozambique	2011	DHS	55.3 (51.2–59.1)	22.6 (20.5–24.8)	12.4 (10.9–14.1)	18.6 (16.6–20.7)	46.1 (42.6–49.6)	6.4 (5.3–7.7)	5.3 (4.4–6.4)	3.5	13.3 (10.2–16.3)
Namibia	2013	DHS	84.0 (81.3–86.4)	33.7 (30.1–37.4)	42.9 (40.0–45.8)	32.9 (29.3–36.6)	78.0 (75.4–80.3)	23.1 (20.2–26.2)	22.6 (19.8–25.7)	1.5	10.3 (3.6–16.8)
Nigeria	2013	DHS	63.0 (60.0–66.0)	35.7 (33.2–38.2)	10.5 (9.3–11.8)	26.1 (23.9–28.4)	55.6 (52.5–58.6)	6.1 (5.1–7.3)	4.9 (4.0–5.9)	5.4	21.3 (18.1–24.4)
Rwanda	2010	DHS	76.9 (73.7–79.7)	60.8 (58.0–63.5)	3.7 (2.9–4.7)	47.2 (43.9–50.5)	55.5 (51.4–59.3)	3.2 (2.4–4.2)	2.6 (1.9–3.6)	18.1	44.6 (40.3–48.6)
Senegal	2014	DHS	80.9 (76.5–84.6)	43.4 (37.1–50.0)	17.9 (13.5–23.2)	39.3 (33.2–45.9)	77.1 (72.0–81.4)	12.0 (8.3–17.1)	11.3 (7.7–16.6)	3.5	28.0 (16.6–38.3)
Sierra Leone	2013	DHS	64.9 (60.0–69.3)	12.7 (10.9–14.8)	9.1 (7.2–11.4)	10.9 (9.2–13.0)	55.5 (50.6–60.2)	3.8 (2.9–5.1)	3.2 (2.4–4.4)	3.4	7.7 (4.8–10.6)
Swaziland	2010	MICS	67.6 (63.6–71.2)	53.7 (50.3–57.1)	30.8 (28.2–33.5)	37.5 (34.0–41.1)	62.1 (58.1–65.8)	20.8 (18.3–23.4)	16.2 (13.9–18.7)	2.3	21.3 (15.3–27.2)
Togo	2013–2014	DHS	64.3 (59.6–68.7)	15.2 (13.2–17.4)	10.1 (8.7–11.8)	12.8 (11.0–14.9)	58.1 (53.6–62.3)	4.1 (3.3–5.1)	3.7 (2.9–4.7)	3.5	9.1 (6.3–11.9)
Uganda	2011	DHS	71.4 (67.7–74.7)	17.2 (14.7–19.9)	7.6 (6.0–9.4)	13.4 (11.0–16.3)	45.7 (42.3–49.0)	3.6 (2.6–4.9)	2.6 (1.8–3.9)	5.1	10.8 (7.1–14.5)
Zambia	2014	DHS	66.7 (63.9–69.3)	28.8 (25.8–32.0)	14.7 (12.8–16.5)	23.3 (20.6–26.1)	60.5 (57.7–63.2)	8.8 (7.2–10.7)	8.2 (6.6–10.0)	2.8	15.1 (10.5–19.5)
Zimbabwe	2014	MICS	78.8 (76.7–80.9)	36.5 (34.3–38.8)	12.0 (10.7–13.3)	31.7 (29.5–33.9)	69.1 (66.9–71.2)	8.0 (7.0–9.2)	7.7 (6.7–8.8)	4.1	24.0 (20.7–27.2)
Total for SSA			**64.5** (60.8–68.0)	**25.7** (23.1–28.6)	**9.3** (7.9–10.9)	**19.7** (17.4–22.3)	**53.6** (50.0–57.1)	**5.0** (4.2–6.1)	**4.2** (3.3–5.5)	**4.7**	**15.5** (11.9–19.0)

* No data on collection time for water were available for Central African Republic or Chad.

The frequency ratio of countries’ coverage for the combined MDG indicator compared to the combined SDG indicator ranged from 1.4 in Namibia, the country with the highest level of combined SDG access to 19 in Liberia, the country with the lowest level of combined SDG access. The majority of countries have MDG:SDG access ratios of around 5:1 or less, with five countries having exceptionally high ratios of 7.5 and over: Burundi (8.1), Democratic Republic of the Congo (8.4), Ethiopia (7.8), Liberia (19.0) and Rwanda (18.1).

### 3.2 Combinations of access to water, sanitation and hygiene

**Fig 1:**
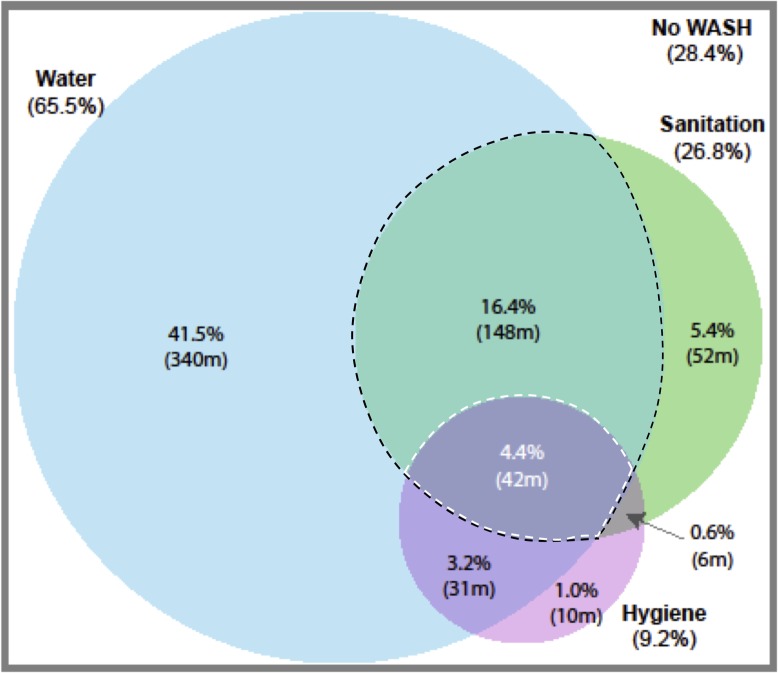
Regional population proportions and estimated numbers in millions of access to differing service levels. Population estimates made using United Nations, Department of Economic and Social Affairs, Population Division (2015). World Population Prospects: The 2015 Revision, custom data acquired via website. Black dashed outline highlights combined MDG indicator and white dashed outline highlights combined SDG indicator without adjusting for water collection time

**Fig 2:**
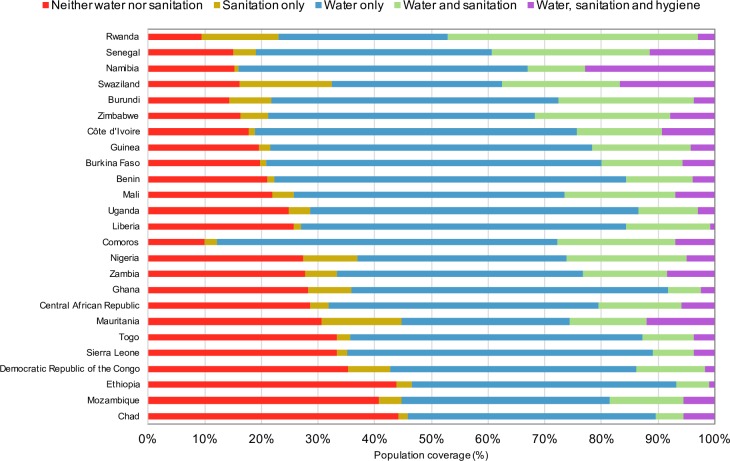
Population using different combinations of WASH facilities by country in sub-Saharan Africa (%).

The most common WASH access scenario in SSA is improved water without improved sanitation or hygiene facilities (Fig 1) with almost half (41.5%) of the population in this situation. This is true for almost all individual countries, too. After this, approximately a sixth (15.4%) have improved water and sanitation without improved hygiene. However, less than 5% (4.4%) of the SSA population have access to improved water, sanitation and hygiene, or combined SDG access. Approximately a third of the population (28.4%) have no facilities. There are substantial variations between countries in the proportions of the population using different combinations of WASH facilities (Fig 2).

### 3.3 Urban rural inequalities

**Fig 3:**
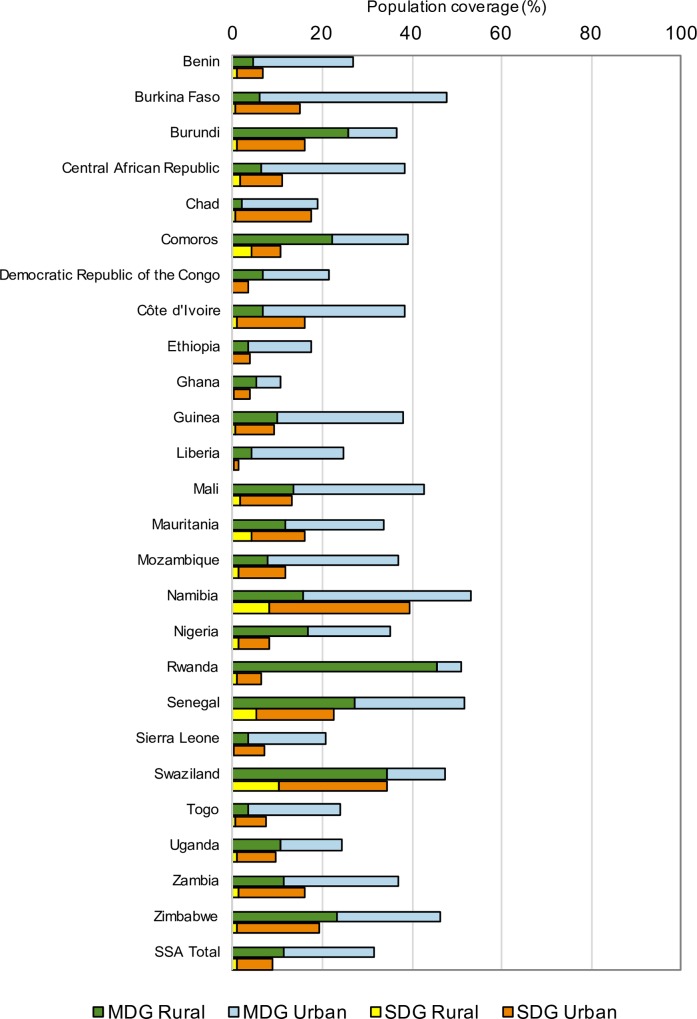
Population coverage of combined MDG and combined SDG access by country, urban and rural populations.

National and regional estimates for urban and rural population levels of combined MDG and combined SDG access are shown in Fig 3 and Table 3. For urban populations the regional estimate for the combined MDG indicator was 31.4%, with national estimates ranging from 10.6% in Ghana to 53.0% in Namibia. For rural populations the regional estimate for combined MDG coverage was 11.4%, with national estimates ranging from 2.0% in Chad to 45.4% in Rwanda. For combined SDG coverage the regional estimate for urban populations was 9.0%, with national estimates ranging from 1.4% in Liberia to 39.7% in Namibia, while for rural populations the regional estimate was 1.0%, with national estimates ranging from 0.0% in Ethiopia to 11.0% in Swaziland.

**Table 3:** Urban and rural coverage of combined MDG and combined SDG indicators and their frequency ratios and percentage point differences.

Country	Combined MDG Indicator				Combined SDG Indicator			
	Urban coverage (% (95% CI))	Rural coverage (% (95% CI))	Ratio Rural / Urban coverage	Percentage point difference Urban–rural (95% CI)	Urban coverage (% (95% CI))	Rural coverage (% (95% CI))	Ratio Rural / Urban coverage	Percentage point difference Urban–rural (95% CI)
Benin	26.9 (24.8–29.1)	4.7 (4.1–5.5)	0.2	22.1 (19.3–25.0)	6.9 (5.9–7.9)	0.9 (0.7–3.8)	0.1	6.0 (4.8–7.2)
Burkina Faso	47.9 (44.4–51.4)	5.9 (5.0–7.0)	0.1	42.0 (37.4–46.4)	15.1 (12.5–18.2)	0.6 (0.4–5.5)	0.0	14.5 (11.6–17.8)
Burundi	36.4 (28.1–45.6)	25.9 (23.5–28.4)	0.7	10.5 (-0.3–22.1)	15.9 (10.4–23.6)	1.1 (0.7–3.4)	0.1	14.9 (8.7–22.9)
Central African Republic	38.5 (35.4–41.7)	6.4 (4.9–8.2)	0.2	32.1 (27.2–36.8)	11.0 (8.7–13.8)	1.8 (1.2–5.9)	0.2	9.2 (6.1–12.6)
Chad	31.2 (27.6–35.0)	2.2 (1.5–3.1)	0.1	29.0 (24.5–33.4)	17.4 (15.1–20.)	0.7 (0.4–5.4)	0.0	16.7 (13.9–19.6)
Comoros	39.0 (33.4–44.9)	22.2 (18.7–26.1)	0.6	16.8 (7.3–26.2)	10.7 (7.8–14.5)	4.1 (2.9–6.3)	0.4	6.6 (2.0–11.6)
Côte d'Ivoire	38.2 (32.6–44.1)	6.9 (5.6–8.4)	0.2	31.3 (24.2–38.5)	16.0 (12.4–20.4)	0.9 (0.5–9.3)	0.1	15.1 (10.8–20.0)
Democratic Republic of the Congo	21.5 (17.6–26.0)	6.9 (5.0–9.3)	0.3	14.7 (8.4–20.9)	3.4 (2.4–4.8)	0.1 (0.0–1.6)	0.0	3.3 (2.2–4.7)
Ethiopia	17.4 (13.3–22.6)	3.5 (2.7–4.6)	0.2	13.9 (8.7–19.9)	3.7 (1.6–8.3)	0.0 (0.0–0.9)	0.0	3.7 (1.5–8.3)
Ghana	10.6 (8.8–12.7)	5.2 (4.3–6.3)	0.5	5.4 (2.4–8.4)	4.0 (3.0–5.3)	0.3 (0.2–2.4)	0.1	3.7 (2.5–5.2)
Guinea	38.0 (33.5–42.6)	9.9 (7.9–12.4)	0.3	28.0 (21.1–34.7)	9.2 (6.8–12.1)	0.5 (0.2–4.1)	0.1	8.7 (5.7–11.9)
Liberia	24.6 (20.1–29.6)	4.2 (2.9–5.9)	0.2	20.4 (14.3–26.6)	1.4 (0.8–2.4)	0.1 (0.0–0.8)	0.1	1.3 (0.5–2.4)
Mali	42.5 (38.6–46.5)	13.6 (11.3–16.3)	0.3	28.9 (22.3–35.2)	13.4 (11.0–16.2)	1.6 (1.1–6.8)	0.1	11.8 (8.7–15.1)
Mauritania	33.9 (30.7–37.2)	11.7 (9.7–13.9)	0.3	22.2 (16.8–27.5)	15.9 (13.7–18.4)	4.2 (3.2–11.5)	0.3	11.7 (8.3–15.2)
Mozambique	36.9 (33.6–40.3)	7.7 (6.6–9.0)	0.2	29.2 (24.6–33.8)	11.8 (10.0–13.8)	1.5 (1.1–5.3)	0.1	10.3 (7.9–12.7)
Namibia	53.0 (47.6–58.2)	15.5 (13.5–17.9)	0.3	37.4 (29.8–44.8)	39.5 (34.9–44.3)	8.0 (6.6–22.6)	0.2	31.5 (25.3–37.6)
Nigeria	35. (32.5–37.7)	16.9 (15.1–18.9)	0.5	18.1 (13.7–22.5)	8.3 (7.0–9.8)	1.3 (0.9–4.9)	0.2	7.0 (5.2–8.9)
Rwanda	51.0 (45.1–56.8)	45.4 (43.3–47.6)	0.9	5.5 (-2.5–13.5)	6.4 (4.7–8.8)	0.8 (0.6–2.6)	0.1	5.6 (3.6–8.2)
Senegal	51.5 (45.1–58.1)	27.2 (21.5–33.9)	0.5	24.3 (11.2–36.7)	17.4 (12.6–23.5)	5.3 (2.8–11.3)	0.3	12.1 (2.9–20.7)
Sierra Leone	20.6 (17.5–24.0)	3.4 (2.7–4.3)	0.2	17.2 (13.2–21.3)	7.1 (5.3–9.4)	0.2 (0.1–3.2)	0.0	6.9 (4.8–9.4)
Swaziland	47.3 (42.4–52.3)	34.4 (31.4–37.5)	0.7	13.0 (4.9–21.0)	34.5 (30.0–39.2)	10.4 (8.8–16.2)	0.3	24.1 (17.8–30.5)
Togo	24.2 (21.6–27.0)	3.7 (2.6–5.2)	0.2	20.5 (16.3–24.4)	7.6 (6.2–9.2)	0.5 (0.3–3.7)	0.1	7.1 (5.2–8.9)
Uganda	24.3 (20.1–29.1)	10.6 (8.6–13.0)	0.4	13.7 (7.1–20.5)	9.7 (6.6–14.0)	0.8 (0.5–2.6)	0.1	8.9 (5.3–13.5)
Zambia	36.8 (32.5–41.4)	11.4 (10.2–12.8)	0.3	25.4 (19.7–31.2)	15.9 (13.0–19.3)	1.4 (1.0–8.2)	0.1	14.5 (11.0–18.3)
Zimbabwe	46.4 (43.5–49.4)	23.1 (21.3–25.)	0.5	23.4 (18.6–28.1)	19.2 (16.9–21.6)	1.0 (0.8–7.7)	0.1	18.1 (15.6–20.8)
Total for SSA	**31.4** (28.0–35.1)	**11.3** (9.8–13.2)	**0.4**	**20.0** (14.8–25.3)	**8.8** (7.1–11.2)	**0.9** (0.6–4.2)	**0.1**	**7.9** (5.7–10.6)

Relative urban–rural inequality as characterised by the frequency ratio of coverage was greater for combined SDG coverage than combined MDG coverage.

**Fig 4:**
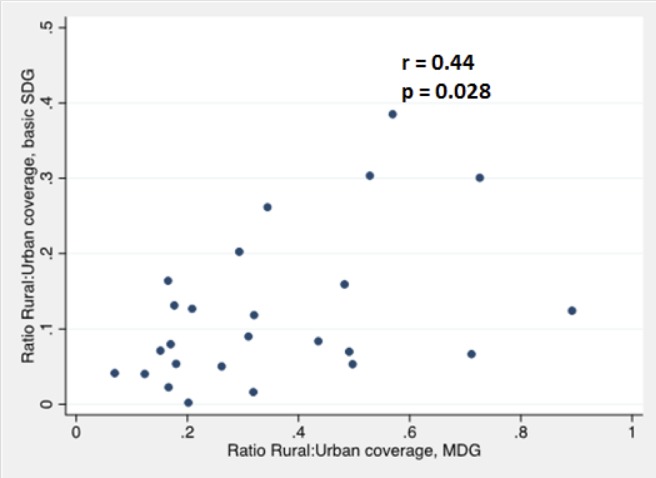
Relationship between ratios of rural:urban coverage for MDG and SDG indicators.

For combined SDG coverage, the regional estimate for urban populations was 8 times greater than for rural populations, whereas for combined MDG coverage, urban populations had three times greater coverage than in rural populations. For rural populations two countries, Ethiopia and Ghana, had estimates that rounded to zero coverage for combined SDG coverage, and only five countries had estimates of over 2%: Senegal (4.1%), Comoros (4.1%), Mauritania (4.2%), Namibia (8.2%) and Swaziland (11.0%). Rural—urban frequency ratios for individual countries for combined MDG coverage ranged from 0.1 in Burkina Faso and Chad to 0.9 in Rwanda, whereas for combined SDG coverage rural—urban frequency ratios ranged from 0.002 in Ethiopia to 0.4 in Comoros. Relative inequality between urban and rural levels of coverage as expressed by the frequency ratio was moderately correlated across the two indicators (r = 0.44, p = 0.028, Fig 4).

**Fig 5:**
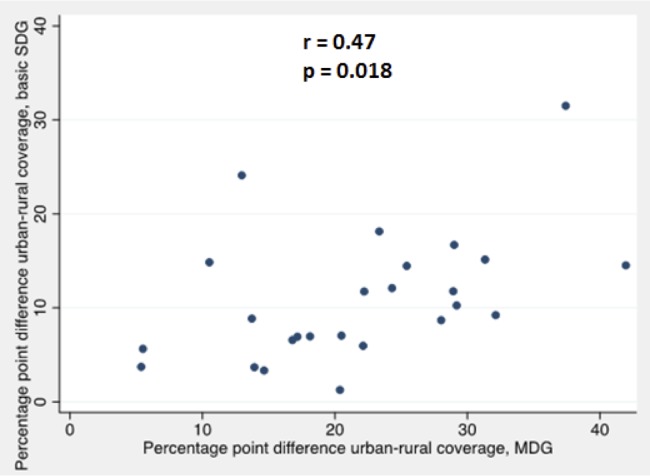
Relationship between percentage point differences between urban and rural coverage for MDG and SDG indicators.

Absolute urban–rural inequality as expressed by the percentage point difference in coverage was greater overall for the combined MDG indicator than the combined SDG indicator, as the overall level of MDG coverage was higher. As a region, rural coverage was 20.0 percentage points lower than urban coverage for the combined MDG indicator, while for the combined SDG indicator it was 7.9 percentage points lower. For individual countries, the percentage point difference between urban and rural populations for the combined MDG indicator ranged from 5.4 in Ghana to 42.0 in Burkina Faso, although two countries, Burundi and Rwanda had no significant difference in urban and rural coverage as the 95% CI for the percentage point difference crossed zero. For combined SDG coverage the percentage point difference ranged from 1.3 in Liberia to 31.5 in Namibia and the difference was significant in all countries. Absolute inequality, as expressed by percentage point difference in urban and rural coverage was also moderately correlated across the two indicators (r = 0.47, p = 0.018, Fig 5).

### 3.4 Wealth inequalities

**Fig 6:**
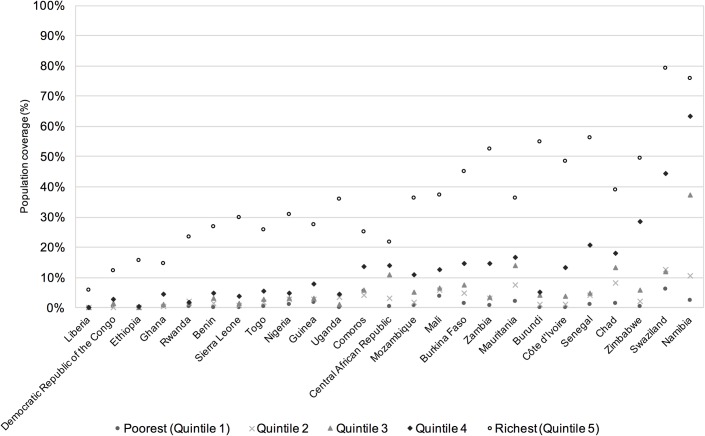
Urban population coverage of combined SDG indicator by country and wealth quintile, ordered by country mean.

**Fig 7:**
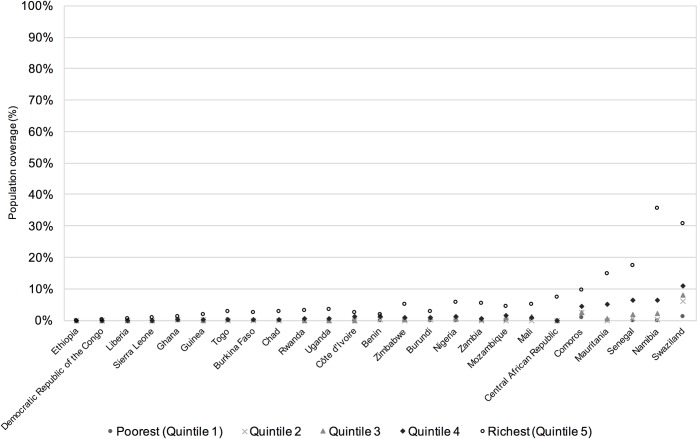
Rural population coverage of combined SDG indicator by country and wealth quintile, ordered by country mean.

Visual examination of the coverage rates across wealth quintiles found that for both indicators the majority of countries showed a top inequality pattern in both urban and rural populations (Figs 6 and 7), indicating that coverage of both combined MDG and combined SDG indicators occurs predominantly in the highest wealth quintile, with very low rates of coverage in the lower quintiles.

For combined SDG coverage, in urban populations most of the countries show a strong top inequality pattern (Fig 6), although some countries have a linear pattern, indicating a more regular increase in coverage as the wealth index increases, e.g. Central African Republic, Swaziland and Namibia. For rural populations (Fig 7) all of the countries show a strong top inequality pattern, although in some cases this is hard to see due to the overall coverage rates being so low. Rates of coverage for the combined SDG indicator are zero for the lowest wealth quintile in five countries’ urban populations: Burundi, Côte D’Ivoire, Ethiopia, Liberia and Sierra Leone, and for eighteen countries’ rural populations. For urban populations, one country, Ethiopia, has zero rates of coverage in the three poorest wealth quintiles. For rural populations, zero rates of coverage extend to the second poorest quintile for twelve countries, to the third poorest quintile for Côte D’Ivoire, and there are five countries where combined SDG coverage is only found in the wealthiest quintile: Central African Republic, Chad, Democratic Republic of the Congo, Ethiopia and Sierra Leone.

**Fig 8:**
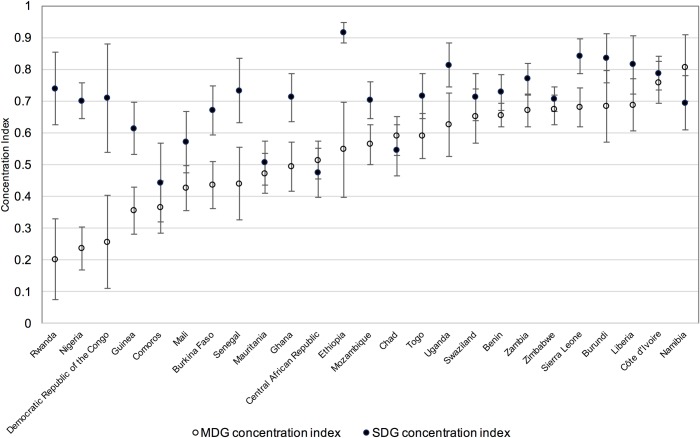
Concentration indices for MDG and SDG coverage in urban populations, ordered by MDG concentration index. Vertical bars show 95% confidence intervals for the concentration indices.

**Fig 9:**
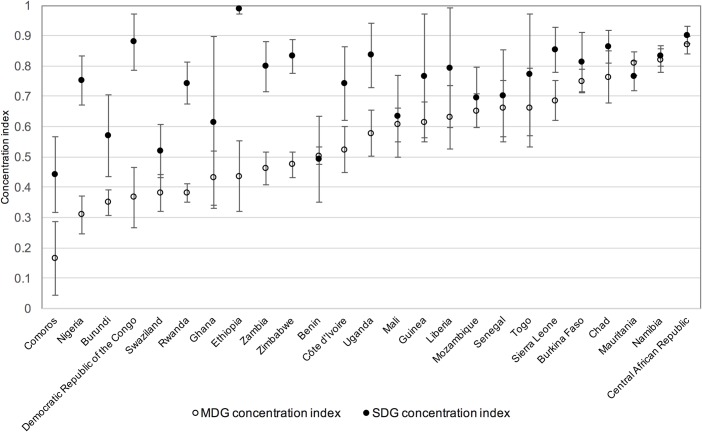
Concentration indices for MDG and SDG coverage in rural populations, ordered by MDG concentration index. Vertical bars show 95% confidence intervals for the concentration indices.

Concentration indices, calculated as a summary measure of inequality and their 95% CI are shown in Figs 8 and 9. Concentration indices take value of zero where there is no wealth-related inequality, negative values indicate higher prevalence of the indicator amongst the poor and positive values indicate higher coverage amongst the rich, with values approaching ±1 indicating a higher degree of inequality in the distribution of the indicator. Concentration indices here are positive and high for both indicators, confirming the inequalities observed in the visual examination of coverage across wealth quintiles. On average, concentration indices are higher–or more unequal—for rural than for urban populations and higher overall for combined SDG coverage than combined MDG coverage.

The mean concentration index (weighted by population) for the combined MDG indicator in urban populations is 0.40, with Rwanda having the lowest concentration index at 0.20 and Namibia the highest at 0.80. For rural populations the mean concentration index for the combined MDG indicator is slightly higher at 0.46, with a range from 0.17 for Comoros to 0.87 for Central African Republic. For urban combined SDG coverage, the mean concentration index is 0.72, ranging from 0.44 for Comoros to 0.92 for Ethiopia, while the mean for rural combined SDG coverage is higher still at 0.81, ranging from 0.44 for Comoros to 0.99 for Ethiopia.

Although the mean concentration index for combined SDG coverage is higher than for combined MDG coverage in both urban and rural populations, in many countries the difference between the concentration indices for the two indicators is not significant at the 95% level. For Benin, Central African Republic, Chad, Liberia, Mali, Mauritania, Namibia, Senegal, Swaziland and Togo there are no significant differences in concentration indices for the two indicators in either urban or rural populations.

For the remaining countries, concentration indices are significantly higher for combined SDG coverage than combined MDG coverage in either the urban or the rural population, or both. Four countries are notable for the concentration index for combined SDG coverage being substantially higher than for combined MDG coverage in both urban and rural populations: Democratic Republic of the Congo, Ethiopia, Nigeria and Rwanda.

## Discussion

### 4.1 Combined access to water, sanitation and hygiene

Access to improved WASH facilities is already known to be low in SSA [1]. In this analysis the estimate for proportion of the population using both improved water and improved sanitation is substantially lower than the separate figures for water and sanitation, and where the combined estimate includes access to improved hygiene as well, access is much lower again. The estimated prevalence of combined MDG coverage over the SSA region was 20%; 6 percentage points lower than the estimate for coverage of improved sanitation (26%), indicating that in this analysis a fifth of those who met the MDG criteria for improved sanitation did not have access to improved water, an estimated 58.9 million people.

The estimate for combined SDG coverage was very low at 4%, indicating that four fifths of those with improved water and sanitation, an estimated 147.8 million people, did not have a facility for handwashing, and a total of 921.6 million people in SSA lacked combined SDG coverage. Ratios of MDG:SDG coverage were high in all countries and were exceptionally high in the countries with the lowest prevalence of combined SDG coverage:–Liberia, Ethiopia, Democratic Republic of the Congo and Rwanda, highlighting that in these countries the prevalence of access to handwashing facilities is much lower than the prevalence of combined MDG coverage. An exception to this is Ghana, which has very low combined SDG coverage (2.4%) but also a relatively low ratio of MDG:SDG coverage at 3.4, as it also has very low rates of combined MDG coverage (8.2%).

In contrast to water and sanitation, hygiene was not included under the MDG targets. As hygiene is included in the targets for the SDGs and the goal is for universal access [2], these results give a first estimate of the progress that is required to achieve the SDG objective of universal access in SSA. Overall, there is a strong correlation in how countries rank on MDG combined versus combined SDG access, but the analysis identified that some countries’ ranks substantially change when the different indicators are considered. Addressing disparities between countries in combined SDG access would require a specific focus on access to handwashing facilities, particularly in cases where levels of access to handwashing facilities are exceptionally low compared to levels of access to improved water and sanitation.

### 4.2 Sub-national inequalities

Access was higher in urban than rural populations for both indicators in all countries included in the analysis. Inequalities of access to both improved water and improved sanitation between urban and rural populations in every country were reported by the JMP, although the gaps have been decreasing [1]. For combined MDG coverage there are some countries that are closer to the ideal of high coverage, low inequality, for example Rwanda (urban coverage 51.0%, concentration index 0.20, rural coverage 45.4%, concentration index 0.38), whereas other countries with high overall coverage such as Namibia (urban coverage 53.0%, concentration index 0.80; rural coverage 15.5%, concentration index 0.82), also have high inequality and very low rates of coverage in the lower wealth quintiles. For combined SDG coverage, as well as having an overall lower coverage rate, relative urban rural inequalities are greater than those for combined MDG coverage, and rural coverage is extremely low for combined SDG coverage in almost all countries. Ethiopia has among the lowest rates of combined SDG coverage in both urban and rural populations and also the highest relative inequality, with concentration indices of 0.92 for urban populations and 0.99 for rural populations.

Absolute urban–rural inequalities are lower for the combined SDG indicator than the combined MDG indicator, reflecting the lower coverage rates overall. Relative wealth-related inequality as measured by concentration indices is high for both indicators, and higher for combined SDG coverage and in rural populations. For the countries that did not have significant differences in concentration indices across the two indicators (Benin, Central African Republic, Chad, Liberia, Mali, Mauritania, Namibia, Senegal, Swaziland and Togo) it appears that access to improved hygiene has a similar distribution across the wealth index to access to improved water and sanitation, so that including the hygiene measure in the indicator does not substantially alter the relative inequality in the distribution, despite absolute levels of coverage being much lower. For countries that had substantially higher concentration indices for combined SDG coverage (Democratic Republic of the Congo, Ethiopia, Nigeria and Rwanda), the distribution of access to handwashing is substantially more unequally distributed towards the rich than is the distribution of access to improved water and sanitation, so that in these cases combined SDG coverage has higher levels of relative inequality, in addition to the lower levels of coverage overall.

Nevertheless, the overall rates of combined SDG coverage are so low in most countries that addressing wealth-related inequality may be of less relevance than addressing the overall rate of access to handwashing facilities in the population.

### 4.3 Limitations

In this analysis we have compared combined MDG access with combined ‘basic’ SDG access. The SDG targets state that services should be ‘safely managed’ and we would expect the estimates of combined ‘safely managed’ services to be considerably lower. The JMP recognises that monitoring this higher service level will require additional information sources for many countries and has been supporting data collection, including direct water quality testing in household surveys and the development of new questions on emptying of onsite sanitation facilities [8]. Household surveys included in our analysis provide information on the location of drinking water sources but no corresponding information on availability and quality. For sanitation, data are currently not available on emptying of onsite sanitation facilities and calculation of the indicator will require integration of administrative data on treatment of wastewater and fecal sludge. Repeating the analysis once these data become available would further elucidate the distance to be travelled to reach the SDGs and the extent of inequalities in access to the higher service level.

Trend data are not available for the handwashing indicator. Our estimates are therefore based on individual surveys from different years and the regional estimates combine surveys from multiple years. The regional estimates appear to be broadly comparable but slightly lower than the modelled estimates reported by the JMP for 2015 with 65% vs 68% and 26% vs 30% respectively for improved drinking water and improved sanitation in sub-Saharan Africa. The lack of trend data means that this analysis does not give information on whether the situation is improving or declining over time.

The JMP MDG improved and SDG basic definitions themselves are a further limitation of the analysis, as it has been recognised that these often do not fully reflect variations in safe water and sanitation access [49–51]. Water sources defined as improved by the JMP often do not supply water considered to be microbially safe by international standards [50]; many otherwise improved sources are faecally contaminated [52], and adjusting JMP progress estimates to account for water quality results in substantially lower estimates [53, 54]. Improved sources also do not indicate reliability or quantity of supply and these affect other behaviours that impact on health, including household water storage and hygiene [55]. People’s ability to access the water supply may also be complex, particularly as neighbourhood sources are classed as improved [56]. Regarding sanitation, the apparent lag in sanitation coverage compared to water may be largely an artefact of the fact that the water target is set at the community level and sanitation at the household level [57]. In addition, the water and sanitation indicators are based on self-reported information and this may overestimate actual use [58]. Observation of a handwashing facility with soap can be poorly correlated with the actual practice of handwashing as measured by structured observation [59].

### 4.4 Implications for SDG monitoring and achievement

SDG targets 6.1 and 6.2 set ambitious global targets for universal access to drinking water, sanitation and hygiene and their achievement will be critical to the realisation of other SDGs. The new indicators (“safely managed services”) address many of the limitations of MDG monitoring by addressing the safety, availability and accessibility of drinking water and the safety of the full sanitation chain. The proposed monitoring framework builds on the MDG “improved” and will be even more challenging to achieve in sub-Saharan Africa.

Overall, our results highlight the great number of people without “basic” household access to water, sanitation and hygiene in sub-Saharan Africa. Our findings suggest that access to hygiene facilities in particular is a major barrier to achieving combined SDG access, reducing coverage in SSA from 19.7% to 4.4%. Handwashing with soap has been found to have a strong effect on reducing diarrhoea transmission [26, 32, 34] and the lack of hygiene facilities may contribute significantly to the relatively high diarrhoeal disease burden in SSA [11]. A priority for SDG monitoring will be to collect standardised information on handwashing and assess trends in coverage for sub-Saharan Africa.

The SDGs maintain separate targets for access to safe and affordable drinking water (target 6.1) and to adequate and equitable sanitation and hygiene (target 6.2). As has been demonstrated by these results, assessing combined coverage at the “basic” level is possible with existing data collection methods and provides important information with regard to household access to combined services. However, despite the benefits of obtaining estimates of combined WASH coverage, there are also risks in using combined indicators, as progress in one area may be masked by a lack of progress in another, as evidenced by the results of this analysis. In combining sanitation and hygiene within one target for the SDGs, the much lower prevalence of handwashing facilities alters the baseline of improved sanitation access substantially and may mean that real progress in improving sanitation facilities is not recognised. This is concerning if it affects motivation and action to improve provision of sanitation facilities where they are not yet in place. It is likely to be beneficial to policy makers to have access to information on *both* separate and combined estimates during the SDG era to provide additional insights on coverage, trends and inequalities and to help with setting national targets.

Our estimates help to quantify the scale of progress required to achieve universal WASH access in low-income countries, as envisaged under the water and sanitation SDG. Monitoring and reporting changes in the proportion of the national population with access to water, sanitation *and* hygiene may be useful in focusing WASH policy and investments towards the areas of greatest need under the SDGs.

## Supporting information

S1 Table Classification of additional categories used in DHS and MICS surveys. (DOCX)
