# Correction: A long way to go - Estimates of combined water, sanitation and hygiene coverage for 25 sub-Saharan African countries

**DOI:** 10.1371/journal.pone.0173702

**Published:** 2017-03-06

**Authors:** Rachel Roche, Robert Bain, Oliver Cumming

The following information is missing from the Funding section: This work was partly supported by the UK Department for International Development through the SHARE research programme consortium.
