# Supplementary material for: A long way to go – Estimates of combined water, sanitation and hygiene coverage for 25 sub-Saharan African countries
Source: PLoS One. 2017 Feb 9;12(2):e0171783. doi: 10.1371/journal.pone.0171783 (PMC5300760; doi:10.1371/journal.pone.0171783)
Supplement: S1 Table — (DOCX) [file pone.0171783.s001.docx]

Table 2. Classification of additional categories used in DHS and MICS surveys

| Survey | Source of drinking water | | Source of water for other purposes^a^ | | Sanitation type | |
| --- | --- | --- | --- | --- | --- | --- |
|  | Improved | Unimproved | Improved | Unimproved | Improved | Unimproved |
| Burundi | ‘Other' classed as improved |  |  |  |  |  |
| Central African Republic | Pump well piped | Pump well non-piped | Pump well piped | Pump well non-piped | Hole latrine with slab |  |
| Mauritania | Puits ou forage a PMH Modern covered well | Modern uncovered well Traditional covered well Traditional uncovered well |  |  |  |  |
| Mozambique | Neighbour's house Borehole with pump |  |  |  |  |  |
| Senegal |  |  |  |  | Latrine with manual flush | Traditional latrine |
| Uganda | Borehole in yard/ plot Public borehole Protected well / spring in yard / plot Protected public spring / well | Unprotected well / spring in yard / plot Unprotected public well / spring |  |  | Covered pit latrine with slab Ecosan | Covered pit latrine no slab Uncovered pit latrine with slab Uncovered pit latrine no slab |

No data available on source of water for other purposes for Benin, Burkina Faso, Burundi, Comoros, Côte d'Ivoire, Democratic Republic of the Congo, Guinea, Liberia, Mali Mozambique, Namibia, Nigeria, Rwanda, Senegal, Sierra Leone, Togo or Zambia
